# Proteomic characterization and evolutionary analyses of zona pellucida domain-containing proteins in the egg coat of the cephalochordate, *Branchiostoma belcheri*

**DOI:** 10.1186/1471-2148-12-239

**Published:** 2012-12-08

**Authors:** Zhongjun Wang, Hua Ye, Xiaoyin Chen, Xi Yang, Yiquan Wang, Liangbiao Chen

**Affiliations:** 1 Key Laboratory of Sustainable Exploitation of Oceanic Fisheries Resources, Ministry of Education, College of Marine Sciences, Shanghai Ocean University, Shanghai, People’s Republic of China; 2 School of Life Sciences, Xiamen University, Xiamen, People’s Republic of China; 3 State Key Laboratory of Molecular Developmental Biology, Institute of Genetics and Developmental Biology, Chinese Academy of Sciences, Beijing 100101, People’s Republic of China; 4 Institute of Genetics and Developmental Biology, Chinese Academy of Sciences, Nan-Yi-Tiao Road #3, ZhongGuanCun, Beijing, 100190, People’s Republic of China

**Keywords:** Amphioxus, Zona pellucida protein, Proteomics, Molecular evolution, Sperm-egg interaction

## Abstract

**Background:**

Zona pellucida domain-containing proteins (ZP proteins) have been identified as the principle constituents of the egg coat (EC) of diverse metazoan taxa, including jawed vertebrates, urochordates and molluscs that span hundreds of millions of years of evolutionary divergence. Although ZP proteins generally contain the zona pellucida (ZP) structural modules to fulfill sperm recognition and EC polymerization functions during fertilization, the primary sequences of the ZP proteins from the above-mentioned animal classes are drastically different, which makes it difficult to assess the evolutionary relationships of ZP proteins. To understand the origin of vertebrate ZP proteins, we characterized the egg coat components of *Branchiostoma belcheri*, an invertebrate species that belongs to the chordate subphylum Cephalochordata.

**Results:**

Five ZP proteins (BbZP1-5) were identified by mass spectrometry analyses using the egg coat extracts from both unfertilized and fertilized eggs. In addition to the C-terminal ZP module in each of the BbZPs, the majority contain a low-density lipoprotein receptor domain and a von Willebrand factor type A (vWFA) domain, but none possess an EGF-like domain that is frequently observed in the ZP proteins of urochordates. Fluorescence *in situ* hybridization and immuno-histochemical analyses of *B. belcheri* ovaries showed that the five BbZPs are synthesized predominantly in developing eggs and deposited around the extracellular space of the egg, which indicates that they are *bona fide* egg coat ZP proteins. BbZP1, BbZP3 and BbZP4 are significantly more abundant than BbZP2 and BbZP5 in terms of gene expression levels and the amount of mature proteins present on the egg coats. The major ZP proteins showed high polymorphism because multiple variants are present with different molecular weights. Sequence comparison and phylogenetic analysis between the ZP proteins from cephalochordates, urochordates and vertebrates showed that BbZP1-5 form a monophyletic group and share no significant sequence similarities with the ZP proteins of urochordates and the ZP3 subtype of jawed vertebrates. By contrast, small regions of homology were identifiable between the BbZP and ZP proteins of the non-jawed vertebrate, the sea lamprey *Petromyzon marinus*. The lamprey ZP proteins were highly similar to the ZP1 and ZP2 subtypes of the jawed vertebrates, which suggests that the ZP proteins of basal chordates most likely shared a recent common ancestor with vertebrate ZP1/2 subtypes and lamprey ZP proteins.

**Conclusions:**

The results document the spectra of zona pellucida domain-containing proteins of the egg coat of basal chordates. Particularly, the study provides solid evidence for an invertebrate origin of vertebrate ZP proteins and indicates that there are diverse domain architectures in ZP proteins of various metazoan groups.

## Background

Almost all metazoan eggs are surrounded by a proteinaceous matrix that is referred to as the zona pellucida (ZP) in mammals and the vitelline coat (VC) in non-mammals. ZP/VC proteins play important roles in fertilization and provide a protective barrier for oviparous animals, such as the amphioxus, fishes and amphibians. The family of ZP proteins is characterized by a conserved protein-protein interaction module, the ZP module [1-3]. The ZP module can be divided into two related domains, ZP-N and ZP-C, and the latter domain associates with the external hydrophobic patch (EHP) [4]. The three-dimensional structure of the mammalian ZP3 shows that the EHP lies at the interface between the ZP-N and ZP-C domains, which are connected by a long loop that carries a conserved O-glycan important for sperm binding [5-7]. The dissociation of EHP from the ZP-C domain allows the ZP proteins to polymerize on the surface of oocytes, thus forming the extracellular coat [5,6,8].

ZP proteins have been characterized from diverse metazoan taxa, including many major groups of jawed vertebrates, urochordates and molluscs. In mammals, the ZP proteins are a family of 3–4 genes divided into 3 subtypes [9]. However, the lower vertebrates, including fish [10,11], birds [12] and amphibians [13-17], typically contain a greater number of ZP genes and subtypes [18].

**Figure 1:**
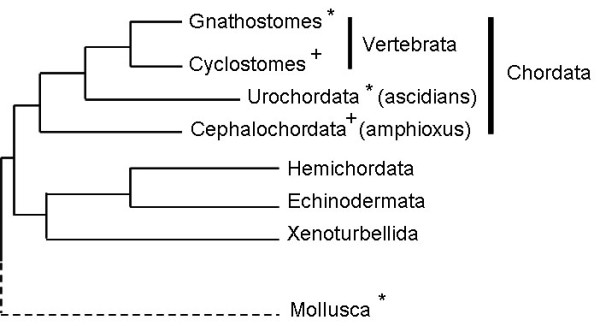
**A cladogram showing the phylogenetic relationship of phyla and subphyla in Deuterostoma and the remote relationship of the protostoma with the phylum Mollusca (dashed lines).** The clades denoted with ‘*’ are the animal groups from which egg coat ZP proteins have been previously identified. The clades denoted with ‘+’ are the subphyla investigated in this study. Figure modified from Bourlat et al., 2006 [28].

The observation that ZP proteins are the major constituents of the vitelline coats of the abalones, an invertebrate group belonging to the protostome phylum Mollusc, which is evolutionarily distant from vertebrates (Figure 1), raised the possibility that the co-option of ZP proteins to form the structural basis of gamete recognition could be a wide-spread phenomenon in metazoan evolution [19,20]. On the one hand, both vertebrate and abalone ZP proteins form monophyletic clades that are distinct from one another, which might suggest independent recruitment of ZP domain-containing proteins rather than a common origin [19]. On the other hand, egg coat ZP proteins rapidly evolve because of positive selection [19]; therefore, it is difficult to infer correct phylogenetic relationships of the invertebrate and vertebrate egg coat ZPs even they had possessed a common ancestor. The recent identification of ZP proteins acting as sperm recognition molecules on the vitelline coats of urochordates (Figure 1), the closest relatives to vertebrates, slightly bridged the gap in the evolutionary landscape of egg coat ZP proteins between the low invertebrates and vertebrates [21-25]. However, it remains unclear whether ZP proteins are also egg coat constituents in the chordate subphylum, Cephalochordata (also known as amphioxus). Recent genome-wide gene comparisons established that Cephalochordata is at the base of chordate phylogeny as the sister group of both urochordates and vertebrates [26-28]. This unique phylogenetic position renders the cephalochordates as an important group in tracing the evolutionary process of the egg coat ZP proteins.

In this study, we characterized the protein composition from the egg coat of a cephalochordate species, *B. belcheri,* by mass spectrometry, from which we identified multiple ZP proteins. We further examined the tissue distribution of the transcripts and mature proteins and observed that they are predominantly expressed in the developing eggs and localized in the cortical granules and extracellular spaces surrounding the eggs. We further identified homologous ZP protein genes from a Cyclostome, *Petromyzon marinus,* to trace the evolutionary relationship of the amphioxus ZP proteins with those of vertebrates. A sequence comparison of the ZP domains among the ZP proteins of gnathostome and cyclostome vertebrates, urochordates and cephalochordates showed that cephalochordate egg coat ZP proteins shared higher sequence similarities with vertebrates than urochordates and reliably suggested a distant homology between the cephalochordate and vertebrate ZPs. Therefore, the chordate egg coat ZP proteins might have a common origin deeply rooted in the lower invertebrates.

## Results

### SDS-PAGE analyses of proteins from unfertilized and fertilized *B. belcheri* egg coats

**Figure 2:**
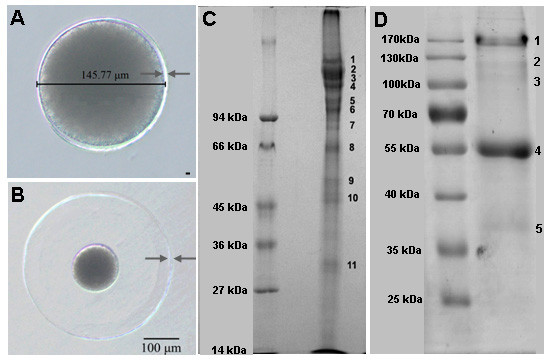
**(A-B). The unfertilized (top) and fertilized (bottom) *B. belcheri *eggs with scale bars indicating the size and elevation of the egg coat before and after fertilization.** The arrows indicate the egg coats that are the subjects of this study. (**C**) and (**D**) show the protein fractions resolved from the unfertilized (panel C) and the fertilized (panel D) egg coat extracts by the SDS-PAGE gel electrophoresis. The gel slicing schemes used for LC-MS/MS analyses are denoted on the right side of each lane. Approximately 20 μg of egg coat extract were loaded on each lane.

A mature and unfertilized *B. belcheri* egg has a diameter of approximately 146 μm and is surrounded by a smooth and round egg coat (EC) layer that is ~6 μm thick (Figure 2A). After fertilization, the EC quickly elevates, which expands the size of the fertilized egg to approximately 400 μm in diameter while leaves the cytoplasmic area unchanged (Figure 2B). However, the thickness of the fertilized egg EC is not significantly reduced during the expansion (Figure 2B), partially because of the discharge of stored EC proteins from the cortical granules and their incorporation into the expanding egg coat. The proteins from the unfertilized and fertilized egg ECs were subjected to SDS-PAGE analysis. Figure 2C shows that proteins in the unfertilized egg extracts could be separated into multiple bands with the major bands estimated to range from ~30 kDa to above 100 kDa. By contrast, protein extracts from fertilized ECs showed fewer and more obviously separated bands with the major band(s) clustered at 55 kDa and some minor ones at 170 kDa, 120 kDa, 110 kDa and 36 kDa (Figure 2D).

### LC-MS/MS identification of zona pellucida domain-containing proteins from the egg coats

To identify the proteins present in the unfertilized egg ECs, the gel containing the electrophoretically separated EC proteins was sliced into 11 pieces (Figure 2C) and subjected to LC-MS/MS analysis. Through a search of the newly derived proteome from the *B. belcheri* entire genome sequencing project (the draft genome of *Branchiostoma belcheri*: http://mosas.sysu.edu.cn/genome/), we identified a total of 1247 unique hits out of the 11 sets of mass spectrometry measurements that met the criteria set in the Sequest software. These proteins were searched against the Pfam 26.0 database, from which we identified five proteins containing the zona pellucida domain (Table 1). These ZP proteins were named BbZP1, BbZP2, BbZP3, BbZP4 and BbZP5 based on the sequential order that they first appeared in the gel slices in the mass spectrometry analyses. Among these proteins, BbZP1 and BbZP3 appeared in 7 and 3 gel slices, respectively (Table 1, the middle panel), which suggests that the 2 proteins may be in multiple forms with different molecular weights in the unfertilized egg ECs.

**Table 1:** **Zona pellucida domain**-**containing proteins identified from B**.**belcheri unfertilized and fertilized egg coats by LC**-**MS**/**MS**

	**Protein ID**	**mature/unfertilized**		**fertilized**	
		**slice no.**	**spectral count**	**slice no.**	**spectral count**
		#1	234		
		#2	50	#1	14
		#3	55	#2	247
BbZP1	304690_PRFO	#4	9	#3	181
		#5	90	#4	42
		#6	8	#5	56
		#8	4		
BbZP2	093040_PFFO	#5	2	#4	38
				#1	60
		#6	5	#2	104
BbZP3	304470_PRFO	#7	23	#3	55
		#8	3	#4	397
				#5	197
				#1	239
				#2	89
BbZP4	001050_PFFO	#10	3	#3	99
				#4	36
				#5	101
BbZP5	093130_PFFO	#11	3	#4	20

To reduce the contamination from the cytoplasmic proteins and to enrich the egg coat protein components for the mass spectrometry analysis, we purified the ECs from the fertilized eggs, which are well separated from the cytoplasmic mass after egg coat expansion. These EC extracts were separated by PAGE and sliced into 5 pieces for LC-MS/MS analysis (Figure 2D). Consistent with what was observed from the unfertilized egg ECs, we identified only the same 5 ZP domain containing proteins from the fertilized egg ECs (Table 1, the right panel), which suggests that the 5 BbZPs compose the complete set of ZP components present in *B. belcheri* ECs. In addition, similar to situations observed in the unfertilized egg samples in which variants of BbZP1 and BbZP3 appeared in multiple gel slices, three BbZPs (BbZP1, BbZP3 and BbZP4) were detected in all 5 gel slices subjected to mass spectrometry in the fertilized egg samples (Table 1, right panel), which suggests the presence of molecular variants with different molecular weights from these gene products.

The spectral counts observed in the mass spectrometry analysis have been suggested to approximately quantify the abundance of each protein in a sample [29-31]. Among the five identified ZP proteins, BbZP1, BbZP3 and BbZP4 showed much higher spectral counts than BbZP2 and BbZP5, which suggests that these three proteins are the major ZP protein types constituting the fertilized egg coat and unfertilized eggs (Table 1). In addition to the zona pellucida domain-containing proteins, a number of proteins were detected in the fertilized egg ECs with spectral counts greater than 10 but fewer than those of BbZPs (Additional file 1: Table S1). These proteins include a multiple EGF-like domain protein, three vitellogenins, one zonadhesin-like protein, one melanotransferrin-like protein, a matrilin and an apolipoprotein B-like protein. A recent study showed that in the ascidian *Halocynthia roretzi*, vitellogenin is a component of the vitelline coat and participates in fertilization as the egg-coat binding partner of sperm proteases [32]. In addition, an apolipoprotein B-like protein has been demonstrated to reside both on the VC and in the egg cytoplasm of the ascidian *C. intestinalis*[22]. Therefore, a few other proteins are commonly present in the ECs of cephalochordates and urochordates in addition to multiple ZP proteins.

### Characterization of the *B. belcheri* ZP genes

We performed RT-PCR and 5’ and 3’ RACE using various primer sets and then sequenced the RT-PCR products to obtain the full-length coding sequences of the five ZP domain-containing proteins. The full-length cDNA of each ZP gene was obtained by piecing together the overlapping fragments. The translated proteins from the full-length BbZP1, BbZP2, BbZP3, BbZP4 and BbZP5 transcripts are 927, 697, 687, 891 and 673 amino acids, respectively. The sequence alignment of the 5 ZP proteins is shown in Additional file 2: Figure S1.

**Figure 3:**
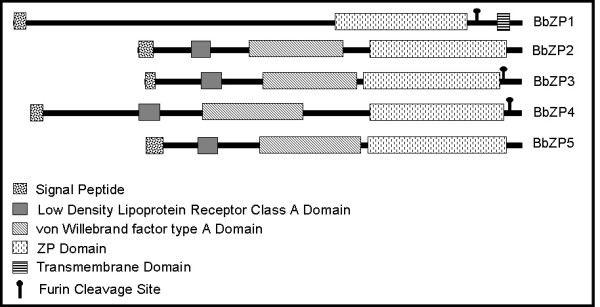
**Diagrams showing the domain architecture of the five *B. belcheri *ZP proteins(see Additional file****2:****Figure S1 for aligned amino acid sequences).** The corresponding nucleotide sequences were deposited in NCBI GenBank under the accession numbers JX046160-JX046164. The domains were annotated using the SMART website ( http://smart.embl-heidelberg.de).

We searched for structural domains within the five ZP proteins in the SMART website ( http://smart.embl-heidelberg.de), and the results are shown in Figure 3. In addition to the ZP module observed in each protein, all BbZPs contain a signal peptide for extracellular secretion. We also identified a low density lipoprotein receptor A (LDLa) domain and a von Willebrand Factor type A (vWFA) domain in BbZP2, 3, 4 and 5, but none in BbZP1. BbZP1 contains a transmembrane domain near its C-terminal that is not detected in other BbZPs. BbZP1, 3, and 4 have a consensus furin cleavage site (CFCS) with four consecutive, positively charged amino acid residues (underlined in Additional file 2: Figure S1). In rare cases, the vWFA domain is also observed to be present in VC proteins of the urochordate *Ciona intestinalis*[22]. Sequence alignments of vWFA domains between BbZPs and those of *C. intestinalis* showed little sequence similarity except at the positions where the vWFA-characteristic amino acids reside (Additional file 3: Figure S2). Notably, the EGF-like domain, which is abundantly present in members of the ZP protein family in urochordates [22,23], is absent in the BbZP proteins.

The above data revealed significant discrepancies between the calculated molecular weights of the predicted full-length BbZPs and the positions each protein migrates on the polyacrilamide gel (i.e., the gel slices in which the proteins were detected by mass spectrometry). Among the 5 BbZPs, the calculated MWs range between ~75 kDa (BbZP5) and ~100 kDa (BbZP1). However, for the unfertilized egg EC sample, protein segments of BbZP1 are abundantly detected in the gel slices that supposedly contained proteins larger than 100 kDa (slices 1–5 of Figure 2C), whereas peptides of BbZP3, BbZP4 and BbZP5 were detected in the gel slices (slices 8–11 of Figure 2C) supposedly containing only proteins with MWs smaller than 75 kDa (Table 1, Figure 2C). The discrepancy is more pronounced in the fertilized EC sample, in which protein fragments of three BbZPs, BbZP1, BbZP3 and BbZP4, are observed in the gel positions (slices 1 and 2 of Figure 2D) for proteins with MWs greater than 120 kDa, whereas the peptides from all 5 BbZPs were detected in gel slices that should have contained proteins with MWs less than 55 kDa (Table 1, Figure 2D). A western blot analysis of the fertilized egg EC extracts, using specific antibodies against the 5 BbZPs, further verified the high polymorphic nature of BbZP1, BbZP3 and BbZP4 (Additional file 4: Figure S3).

Egg coat ZP proteins are well known to be highly glycosylated [5,10,33], which may contribute to the higher than expected MWs in the SDS-PAGE analysis. To determine how potential glycosylation might have affected the BbZPs, we treated the fertilized egg EC extracts with the enzyme PNGase F, which was specific to cleave the N-linked glycosylation moieties off of the proteins. The overall gel migration pattern of the treated sample remained the same, except that the 55 kDa band in the untreated sample narrowly separated into two 55 kDa bands, the faint 37 kDa band in the untreated sample disappeared, and a smaller (~36 kDa) band appeared (Additional file 5: Figure S4). These results indicate that glycosylation, at least N-glycosylation, is not the major factor in the observed aberrant migration patterns of the BbZPs. It is possible that the incomplete disassociation of the BbZP polymers may have resulted in the higher than calculated MWs observed in the polyacrylamide gels.

We checked whether alternative splicing of the BbZP transcripts could occur to understand why some BbZPs appeared in the gels with smaller than calculated MWs. Within sets of specific primers of BbZP1 and BbZP4, we identified cDNA variants that were shorter than expected from the full-length cDNAs (Additional file 6: Figure S5), indicating the presence of alternative splicing. The current survey of the BbZP transcripts is not exhaustive, and more alternatively spliced forms of BbZPs will likely be identified if more complete RT-PCR experiments are performed. However, the smaller sized BbZP variants could be a result of proteolysis of the full-length BbZP precursors. For example, the vitelline coat ZP protein, HrVC70, is derived from HrVC120, which is a larger VC ZP protein in the urochordate *H. roretzi*[22,23].

### Tissue specific expression and cellular localization of the BbZPs

**Figure 4:**
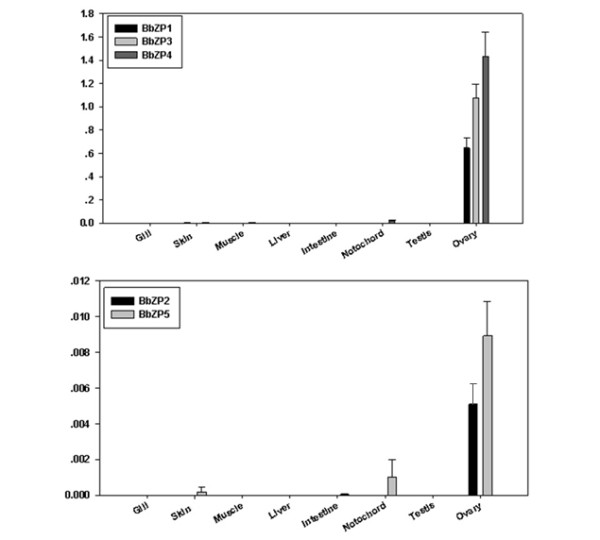
**The expression profile of amphioxus ZP proteins determined by real-time qPCR of RNA from eight tissues of adult *B. belcheri.*** The Y-axis denotes the ratio of ZP proteins to β-actin. The scales of the vertical bars of the two panels are different, and the top panel scale is 100 times greater.

We performed qRT-PCR analyses of total RNAs from the gills, skin, liver, notochord, intestines, testes and ovaries to determine which tissues of *B. belcheri* express the BbZP genes. The results showed that for each gene, ovary tissue showed the most abundant expression (Figure 4). Coinciding with the much higher spectral counts observed in the MS analysis (Table 1), BbZP1, BbZP3 and BbZP4 expressed at levels approximately one-hundred times higher than BbZP2 and BbZP5 in the qRT-PCR measurements, which suggests that BbZP1, 3 and 4 are the major ZP proteins to constitute the egg coat in *B. belcheri*. In addition to the ovary, other tissues, such as the skin and notochord, have low levels (1/100^th^ of that of ovary) of the ZP genes expressed.

**Figure 5:**
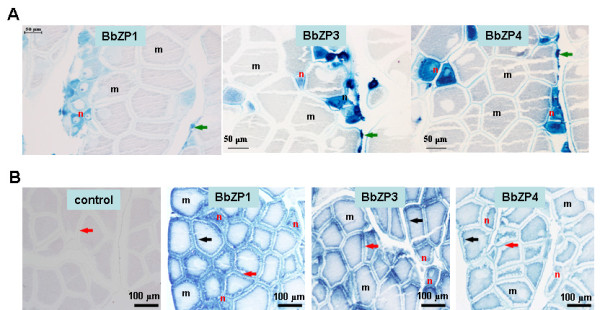
***In situ *hybridization and immunological staining of the three major BbZPs (BbZP1**, **BbZP3 and BbZP4) in ovary sections of adult *B. belcheri *samples. **(**A**). *In situ* hybridization showing the predominant localization of mRNAs of BbZPs in the developing (immature, smaller-sized) oocytes (the cells in blue) in the ovary of the adult amphioxus. No hybridization signal was detected in the developed, larger-sized oocytes (in the light grey colors). Typical immature oocytes are indicated by a red ‘n’, whereas typical mature ones by a black ‘m’. Green arrows indicate the follicle cells with positive signals. (**B**). Immunological localization of the three major ZP proteins in the egg surface (red arrows) and cortical granules (black arrows) in adult amphioxus. Panel a shows the negative control, in which pre-immune rabbit serum was used to replace the first antibody in immunochemical staining. Black ‘m’ and red ‘n’ indicate typical mature and immature oocytes, respectively.

We performed *in situ* hybridization (ISH) using the antisense cRNA probes derived from the three major BbZP genes to determine the ovary cells that BbZP genes are expressed. Strong hybridization signals are detected in small (immature) oocytes, whereas they are absent in large-sized, more mature oocytes (Figure 5A); these findings indicate that the major BbZPs are predominantly expressed in developing oocytes. By contrast, immunohistochemical (IHC) analyses using the specific antibodies against the identical three BbZPs showed more even distributions among the variously sized oocytes across the landscape of the ovarian section. Furthermore, strong IHC signals were observed on the surface of the oocytes and at the areas underneath where the granules localize (Figure 5B). Weak ISH signals of the two minor BbZP types, BbZP2 and BbZP5, were also observed in the developing oocytes (data not shown); however, the detection of IHC signals using the in-house raised antibodies for these two proteins was ambiguous, possibly because of the low amounts of the two ZP types in the egg coats.

### *B. belcheri* ZP proteins showed sequence homology with the non-jawed vertebrate, *Petromyzon marinus*

**Figure 6:**
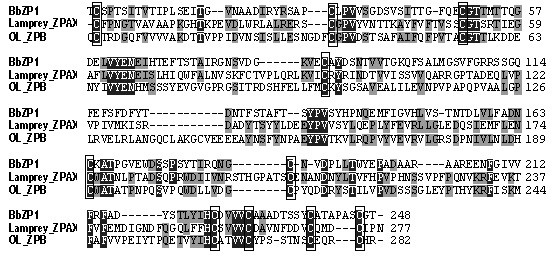
**The alignment of three zona pellucida domains from three ZP proteins from *B****. ****belcheri (BbZP1), P. marinus *(lamprey ZPAX), and *Oryzias latipes *(Ol ZPB) showing sequence homologies between the cephalochordate and vertebrate ZP proteins.** The cysteine residues that are critical for the function and identity of ZP domains are boxed. Positions that are identical in at least two ZP proteins are boxed.

We initially searched the genome of the non-jawed vertebrate *P. marinus* and the predicted proteome databases for BbZP protein homologues to elucidate how the ZP proteins of the basal chordates are evolutionarily related to those of vertebrates. We identified 4 predicted proteins containing the ZP domain (Additional file 7: Figure S6). These proteins are not full-length because of the sequence gaps in the current public version of the *P. marinus* genome. However, when the ZP domains from BbZP1, PmZP1 and OlZPA (representing the ZP proteins from cephalochordates, non-jawed vertebrates and jawed vertebrates, respectively) are aligned, small regions of sequence homology occurs among the three ZP proteins (Figure 6). Considering that cysteine residues are important for the formation of correct ZP domain structures [34] and that the number and location of the cysteine residues define ZP protein subtypes, we examined the distribution of cysteine residues in the three groups of ZP proteins. We observed that cysteines of BbZP proteins are either in the identical positions as or in close proximities of those of vertebrate ZP proteins. In addition, four of the five BbZP proteins (except BbZP4) and all of the vertebrate ZP proteins identified in this study contain 10 cysteines in the ZP domain region with the first 4 cysteines in the ZP-N moiety and 6 in the ZP-C moiety [4], which is a feature of the type II ZP domain according to the mammalian ZP nomenclature [34].

### Phylogenetic analyses showed that ZP genes of the basal chordate form a distinct evolutionary clade and most likely share a recent common ancestor with the lamprey ZP proteins and ZP1/2 subtypes of the high vertebrates

**Figure 7:**
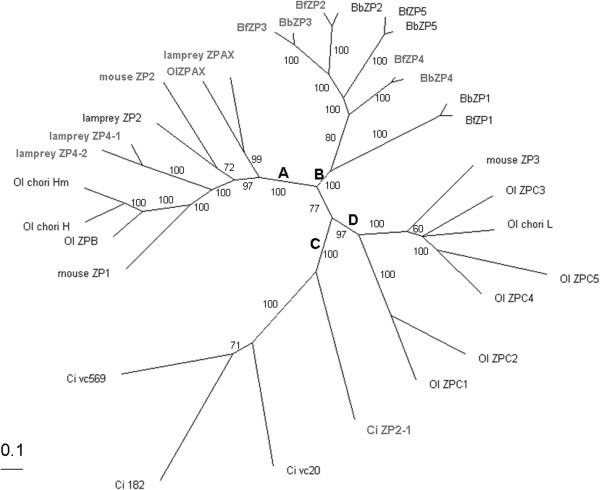
**The evolutionary relationships of ZP proteins from a jawed vertebrate (*O.latipes, *Ol), a non**-**jawed vertebrate (*P****.****marinus, *lamprey), cephalochordates (*B. belcheri *and *B****.****floridae***, **Bb and Bf), and a urochordate (*C. intestinalis, *Ci) deduced by Bayesian analyses.** The tree is drawn to scale with branch lengths in identical units to those of the evolutionary distances used to infer the phylogenetic tree. The posterior probability is shown to the side of each branch. The different ZP protein clades in this figure that are described in the Results section are labeled under the bold letters: **A**,**B**, **C** and **D** respectively.

We gathered entire sets of ZP proteins from species of jawed vertebrates (mammals and Teleost fish, *Mus musculus* and *Oryzias latipes*, respectively), a non-jawed vertebrate (lamprey, *P. marinus*), cephalochordates (*B. floridae* and *B. belcheri*), and a urochordate (*C. intestinalis*) to elucidate the phylogenetic relationships between ZP proteins from the major metazoan lineages. The ZP domain regions of these proteins were identified and aligned. We constructed phylogenetic trees of the ZP domains using Bayes-based and Neighbor-joining (NJ) approaches. The Bayes-based approach yielded trees with higher confidence values for each node than the NJ approach; thus, an unrooted Bayes tree is shown in Figure 7 to demonstrate the evolutionary relationship of the ZPs from the chordates. The ZP proteins from the cephalochordates formed a distinct clade (clade B). Clade B and Clade A, which is composed of the ZP1, ZP2, and ZPAX of the jawed vertebrates and all of the lamprey ZP proteins, appear to share a recent common ancestor. However, the urochordate ZP proteins (Clade C) and ZP3/ZPC of the jawed vertebrates (Clade D) are evolutionarily more distant from the cephalochordate ZP proteins, as judged from the branching lengths (Figure 7). Notably, the lamprey ZP proteins appeared to intercalate with the ZP1, ZP2, and ZPAX subtypes of the jawed vertebrates (i.e., mouse ZP1/lamprey ZP4-1, mouse ZP2/lamprey ZP2 and Ol ZPA/lamprey ZPAX) in the phylogenetic tree (Clade A), which indicates that the egg coat ZP proteins had diversified into distinct subtypes defined in vertebrate ZP nomenclature [35] in early vertebrate evolution. The sequence homology (Figure 6) and phylogenetic relationships deduced from this study (Figure 7) suggest that vertebrate ZP proteins have an invertebrate origin.

## Discussion

Using comprehensive proteomic approaches, we identified 5 proteins containing a zona pellucida domain from *B. belcheri* egg coat extracts (Table 1). We further verified the physical location of these proteins on the egg coat by immunohistochemistry using antibodies raised to specifically target these ZP proteins (Figure 5B). A search for homologous genes in the published *B. floridae* genome revealed that all 5 of the *B. belcheri* genes have *B. floridae* orthologs and that each pair shared more than 80% protein sequence similarity. In addition, the genomic locations and relative orientations of the ZP orthologs from the two species are highly conserved (Additional file 8: Figure S7). In *B. belcheri*, a search of the newly sequenced genome showed that BbZP1 and BbZP3 form a synteny in scaffold 3, BbZP2 and BbZP5 co-localize in scaffold 88, and BbZP4 localizes in a separate scaffold (Scaffold 33). Notably, the genes in Scaffolds 3 and 33 were highly expressed, whereas the ones in scaffold 88 were not. Similarly, in the *B. floridae* genome, the orthologs of BbZP1 and BbZP3 (BfZP1 and BfZP3) are observed in scaffold Bf_V2_271, ~23 kb apart from one another; the orthologs of BbZP2 and BbZP5 (BfZP2 and BfZP5) are linked in tandem in scaffold Bf_V2_78 with ~3 kb between the two. A small difference occurs in the case of BbZP4. Whereas we identified only one BbZP4 gene in the *B. belcheri* genome, two orthologs (BfZP4-1 and BfZP4-2) were observed in *B. floridae*, arranged in a head-to-head orientation in scaffold Bf_V2_243 in the *B. floridae* genome, a scaffold that is distinct to BbZP1/3 and BbZP2/5. A genomic comparison showed that the ZP protein genes identified in this study are common to the genus *Branchiostoma*. The cephalochordates comprise approximately 35 species that are divided into three genera: *Branchiostoma*, *Epigonichthys* and *Asymmetron*[36]. The distribution of these genes among other genera of Cephalochordata requires further study.

In recent years, ZP proteins have been characterized from urochordates, which are phylogenetically the closest relatives of vertebrates. Notably, comparisons of the MS results of *B. belcheri* with those from *C. intestinalis* reveal that the number of ZP proteins identified in this study is significantly less than those observed in *C. intestinalis* (5 vs 11), which suggests that the number of ZP protein genes in the basal chordate species might be fewer than those of the urochordates. In addition to the ZP proteins, there are some non-ZP proteins that might also be constituents of the egg coat, for example, vitellogenin and apolipoprotein B-like protein, which are also the known components of egg coats in urochordates [22,32]. Whereas mammals use ZP proteins as the sole components to compose the zona pellucida matrix that surrounds the egg, the lower chordates appear more variable in selecting the egg coat composition.

ZP proteins have been observed to be the major constituents of egg coats in diverse metazoan groups (Figure 1); however the evolutionary relationship among ZP proteins is not obvious. The identification of ZP proteins as important components of the cephalochordate egg coats has filled a gap in the knowledge regarding chordate ZP proteins and has enabled us to make conjectures regarding the evolutionary processes of ZP proteins in chordates. Both the sequence comparison (Figure 6) and ZP domain tree (Figure 7) indicated that cephalochordate ZP proteins are evolutionary homologues of the lamprey ZP proteins and the ZP1, ZP2 and ZPAX subtypes of the jawed vertebrates, which suggests a common ancestor for the two ZP clades. Therefore, vertebrate ZPs appeared to have an invertebrate origin (i.e., at least began at the base of chordate evolution) rather than an independent recruitment of ZP domain containing proteins. However, the urochordate ZP proteins appeared to be closer to the ZP3/ZPC subtypes of vertebrates (Figure 7). The phylogenetic tree of ZP proteins from the three chordate subphyla indicated that the vertebrate ZP subtypes had two recently separated common ancestors. In addition, except for the ZP modules, which are the common structural domains of all metazoan ZP types, the other domain components of the ZP proteins from cephalochordates, urochordates and vertebrates are different. Von Wallebrand and repetitive EGF domains are found in the egg coat ZP proteins from cephalochordates and urochordates, respectively, which suggests different domain structures may be involved in gamete recognition in the two groups.

The high resolution structures of the mouse ZP3 [7] and ZP-N domains [6] provided an understanding of the structural basis of sperm recognition in mammals. Furthermore, the accumulating evidence suggests that the presence of repeated ZP-N domains in the ZP proteins, in addition to the universal ZP-module, may well be associated with the sperm-binding activity [37]. A recent threading analysis of the VERL repeats in abalone ZP proteins suggested that, similar to their ZP2 counterparts, the VERL repeats most likely adopt a ZP-N fold, as shown by the complete conservation of four cysteine residues within each repeat [38]. The domain architecture of the 5 BbZP proteins (Figure 3) shows that they possess neither the EGF-like domain repeat nor the ZP-N repeats. Most of the lamprey ZP proteins identified in this study are not full-length because of the low coverage of the current available genome. The full-length lamprey ZP2 also lacks an apparent ZP-N domain or an EGF-like domain; therefore, whether or how the BbZP proteins and lamprey ZP proteins function in sperm recognition during fertilization remains an open question that warrants further investigations.

## Conclusions

By comprehensive proteomic analysis, followed by *in situ* hybridization and immunohistochemical analyses, we identified five egg coat ZP proteins from the cephalochordate *B. belcheri*. We also identified four ZP proteins from the jawless vertebrate, the sea lamprey, which are highly similar to vertebrate ZP subtypes. Molecular phylogenetic analyses showed that the *B. belcheri* ZP proteins form a distinct evolutionary clade but are homologous to both the lamprey and vertebrate ZPs in protein sequences. The study traces the evolutionary history of vertebrate sperm-egg recognition molecules to the appearance of basal chordate animals in the metazoan phylogeny.

## Methods

### Animal collection

The amphioxus used in this study were captured from the Xiamen Tong’an coastal waters of the East China Sea during the spawning season (March-October) and transferred in sand mixed with seawater to Shanghai Ocean University for species identification. *B. belcheri* and *B. japonicum* were identified by their morphological traits. The animals were screened for their sex and stage of gonad development, and then stored individually at −80°C for further use. The experimental procedures are followed with the guidelines established by the Ethic Committee for Animal Usage in Research of Shanghai Ocean University where the animal procedures are carried out.

### Egg coat separation

To obtain the egg coats (ECs) of unfertilized eggs, fully developed eggs were surgically separated from the gonad and then shaken in Ca^2+^/Mg^2+^-free artificial seawater until the eggs were fully separated. The eggs were then gently homogenized in 0.2×Ca^2+^/Mg^2+^-free artificial seawater (4 mM EPPS [1-Piperazinepropanesulfonic acid, 4-(2-hydroxyethyl)], pH 8.0, 92 mM NaCl, and 2 mM KCl) containing a protease inhibitor mixture (1 mM phenylmethylsulphonyl fluoride, 10 μg/ml leupeptin) with a Teflon homogenizer. The homogenate was filtered through a nylon mesh (22 um). The EC that remained on the mesh was washed by pipetting, using 0.2×Ca^2+^/Mg^2+^-free artificial seawater containing 0.005% Triton X-100, and further purified manually under a binocular microscope. To obtain the ECs of fertilized eggs, the elevated coats of 20 fertilized eggs were manually peeled and thoroughly rinsed with 0.2×Ca^2+^/Mg^2+^-free artificial seawater at least 5 times. Both the unfertilized and fertilized EC samples were extracted with Laemmli SDS-PAGE sample buffer containing 5% 2-mercaptoethanol and were denatured by boiling for 5 min prior to SDS-PAGE analyses.

### SDS-PAGE separation

The egg coat extracts from unfertilized and fertilized eggs, each with approximately 20 μg proteins, were separated using 15% SDS-PAGE and 1.5 mm-thick gels. After electrophoresis, the gels were stained with Coomassie Blue R250 (Sigma, USA). The gel of the unfertilized eggs was dissected into 11 slices and that of the fertilized eggs into 5 slices (Figure 2) for LC-MS/MS analyses.

### Western blot analysis

The protein concentration of the egg extracts from the fertilized eggs was measured using a BCA protein assay kit (Pierce, Rockford, IL). The egg extracts were subjected to electrophoresis in 12% SDS-PAGE gel. The gels were transferred onto PVDF membranes. The membranes were blocked with 5% (w/v) skimmed milk in TBS overnight at 4°C. The blocked membranes were incubated separately with a primary antibody, namely, polyclonal rabbit Anti-BbZP1, Anti-BbZP3 (diluted 1:3000) or mouse polyclonal antibodies against BBZP2, 4 and 5 (diluted 1:4000) in TBS containing 5% skimmed milk for 1 hr at room temperature. The incubated membranes were then washed with TBS-T 3 times for 15 minutes each. The membranes were incubated with goat anti-rabbit or goat anti-mouse HRP-conjugated secondary antibody (1:5000; Santa Cruz, CA) for 1 hr at room temperature and washed with TBS-T. The blots were visualized by using the Immobilon Western chemiluminescence HRP substrate system (Pierce, Rockford, IL) following exposure to medical X-Ray films (Fuji film, Tokyo, Japan).

### Enzyme digestion, LC-MS/MS analysis and database searching

The dissected, protein-containing gel blocks were subjected to trypsin treatment (0.2 μg, trypsin in 25 mm NH_4_HCO_3_ buffer at 37°C overnight). The digested peptides were extracted by 50% acetonitrile and 5% formic acid at room temperature for 30 mins [39]. The digested products from each gel band were then separated on a Paradigm MS4N Nano/Capillary HS MDLC (Michrom Bioresources, Inc. USA) using a 100 μm x 150 mm C18 reverse phase column. Liquid chromatography was conducted with a linear gradient of buffer A and 5–35% buffer B (50 min) followed by 35–90% buffer B (10 min) and 90% buffer B for 10 minutes at a flow rate of 500 nl/min. Buffer A comprised 0.1% formic acid in a 2% acetonitrile H_2_O solution, and buffer B was 0.1% formic acid in a 98% acetonitrile H_2_O solution. The separated peptides were then subjected to mass spectrometry analysis in a LTQ-MS (Thermol, USA) machine coupled with a Michrome Advanced nanospray apparatus (Microm Bioresources Inc.). The peak list files were generated using the Bioworks software (Applied Biosystems, USA) with the default parameters. The m/z peaks were searched against the predicted protein database (version 1.1) derived from the *B. belcheri* genome project (the draft genome of *Branchiostoma belcheri*: http://mosas.sysu.edu.cn/genome/) using the Sequest software. The parameters were established as follows: Xcorr ≥ 2 for two or three valent ions; Xcorr ≥ 1.5 for one valent ion; Deltacn ≥ 0.1; and at least two nonredundant peptides can be identified in a single protein. The false discovery rate was estimated as <1% using a reversed proteome database as a control. The mass spectrometry data was supplied in the additional file 9: Figure S8. The mass spectrometry data was deposited in the PRIDE database ( http://www.ebi.ac.uk/pride/) under the reference No. 1-20121203-123403.

### Characterization of the full-length transcripts of the zona pellucida domain-containing genes

The total RNA from stage IV *B. belcheri* ovaries was extracted using Trizol (Takara) following the manufacturer’s protocol. Two μg of the total RNA was reverse transcribed by SuperScript® III Reverse Transcriptase (Invitrogen, USA) in 50 mM Tris–HCl (pH 8.3), 75 mM KCl, 5 mM MgCl and 5 mM DTT and zona pellucida domain-containing genes were amplified by polymerase chain reaction (PCR) using pairs of primers designed according to the predicted transcripts of the target genes. To obtain the UTR sequences, both 3’ and 5’ RACE were performed with the SMART RACE kit (TaKaRa Co, Japan) following the manufacturer’s instructions. The PCR products were cloned into the pGEM-easy TA cloning vector and sequenced; the overlapping clones were pieced together to gain the full-length cDNAs of the BbZP genes.

### Animal section preparation

The fully developed gonads from adult *B. belcheri* animals were cut into small fragments (approximately 1 centimeter each) and fixed in 4% paraformaldehyde (PFA) and phosphate buffered saline (PBS, pH 7.4) for approximately 4 hr at room temperature. After dehydration in an ascending series of ethanol and clearing in xylene, the tissues were embedded in paraffin, sectioned transversely at 4–5 μm, and mounted on glass slides (RNase-free), which were precoated with polylysine. Because the sections were used for both *in situ* hybridization and immunohistochemistry, extra caution to avoid RNase contamination was taken.

### *In situ* hybridization

One fragment for each gene was amplified and cloned into the pGEM-T vector (Promega). After verifying the clones by sequencing, the plasmids were purified and cut at one end using the appropriate restriction enzyme. The antisense riboprobe for each gene was synthesized using SP6 or T7 polymerase. The paraffin sections were used to examine their expression patterns as described by Yu and Holland [40].

### Antibody generation and immunohistochemical staining

The divergent regions of BbZP2, 4, and 5 were expressed in bacteria using the pET-28a vector, and the His-tagged recombinant proteins were purified using a nickel bead column according to the instruction manual (Promega) and verified by SDS-PAGE electrophoresis. The purified recombinant proteins were injected into mice to produce polyclonal antibodies, and following the fourth injection (given at one week intervals), the mice were sacrificed for antisera. Anti-BbZP1, Anti-BbZP3 polyclonal antibodies were generated by immunizing rabbit with synthetic peptides. The specificity of the antibodies was verified by a western blot of the specific recombinant protein with bovine serum albumin as a control. Antibody batches with the best specificity were purified. The antibody raising and purification were conducted by Hua-An Biotechnology Inc. in Hangzhou, China. The purified antibodies were used for immunohistochemical staining. The paraffin sections were used to localize the expression regions of the tissues. Immunocytochemical staining was performed with an ABC (avidin-biotin peroxidase complex) kit (Maixin-Bio Co, China) as described by Bočina1 and Saraga-Babić [41].

### Quantitative RT-PCR

The total RNA was extracted from *B. belcheri* gills, skin, muscles, livers, intestines, notochord, testes and ovaries and then reverse transcribed. Two pairs of primers for each ZP gene were designed and tested for the amplification efficacy and specificity by electrophoretic analysis of the PCR products. The primer pair with the best specificity was selected for further use in a Quantitative PCR (Q-PCR). Q-PCR was performed using the SYBR RT-PCR kit in a Bio-Rad CFX 96 (Bio-Rad) machine, and the results were analyzed by CFX Manager software. The PCR was performed using 45 cycles of 95°C for 15 seconds, 72°C for 30 seconds and 60°C for 30 seconds. The PCR reaction for each gene was performed in triplicate with the housekeeping gene beta-actin as the control.

### Phylogenetic analysis

The representative ZP genes from the urochordate *Ciona intestinalis*, the teleost fish *Oryzias latipes*, and the rodent *Mus musculus* were collected from NCBI GenBank entries. The teleost fish ZP genes were used as queries to search against the predicted lamprey cDNA databases (version *Petromyzon_marinus*_7.0), and transcripts with significant similarity (e-value ≤ 1e-20) were identified as ZP protein homologues. The ZP domain region of each ZP protein was delineated by searching against the SMART database [42]. The ZP domain regions were aligned using t-coffee [43]. A Bayesian inference of phylogeny was performed using MrBayes 3.2.1 (John P. Huelsenbeck and Fredrik Ronquist, MRBAYES: Bayesian inference of phylogenetic trees. Bioinformatics 17:754–755). The model selected by MrModeltest 2.3 [44] according to AIC criterion was GTR+I+G. The tree was displayed using the Treeview software.

## Abbreviations

ZP: Zona Pellucida; EC: Egg coat; EGF: Epithelial growth factor; vWFA: Von Willebrand factor type A.

## Competing interests

The authors declare that they have no competing interests.

## Authors’ contributions

Conceived and designed the experiments: LBC YW QX GL. Performed the experiments: QX GL ZW LXC XC XY. Analyzed the data: LBC HY. Wrote the paper: LBC. Contributed to the final version of the manuscript: LBC QX LXC. All authors read and approved the final manuscript.

## Supplementary Material

Additional file 1 **Table S1.** Other non-ZP proteins identified from *B. belcheri* fertilized egg coats. Click here for file

Additional file 2 **Figure S1.** The predicted amino acid sequences and the alignments of the five *B. belcheri* ZP proteins. The gray scale indicates the various numbers (3 to 5) of identical residues in the 5 aligned sequences. The cysteine residues that define the zona pellucida domains are boxed. Except for BbZP4, the other BbZP proteins contained 10 positionally conserved cysteine residues, suggesting a type II ZP domain. Regions with a double underline indicate domains of LDLa; those with the single underlines indicate vWFA domains, and the bold lines indicate the ZP modules. Click here for file

Additional file 3 **Figure S2.** The amino acid sequence alignment of the von Willebrand factor type A (vWFA) domains from the BbZPs (BbZP2, 3 4, and 5) and two ZP proteins of *Ciona Intestinalis* (XP_002120027.1 and XP_002127158.1). Click here for file

Additional file 4 **Figure S3.** Western blot analysisof the egg coat extracts of fertilized B. belcheri eggs. High levels of size polymorphism are detected in the three major BbZPs, BbZP1, 3 and 4, whereas fewer bands are visible for the minor ZPs as well as BbZP2 and 5 under identical conditions. The data correspond well with those obtained from the mass spectrometry assays. The distinctive banding patterns of the individual ZPs sggests that the antibodies are specific to the individual ZP proteins. Click here for file

Additional file 5 **Figure S4.** Eggcoat extracts of fertilized *B. belcheri* eggs digested with PNGase F. Approximately 30 μ g of the egg coat extracts were denatured by heating the solution to 100°C for 10 minutes in a buffer containing 50 mM Sodium phosphate (pH 7.5), 0.2% SDS , 10 mM 2.mercaptoethanol. After cooling the room temperature, 2 units of PNGase F (Sigma USA) were added. The digestion was performed at 37°C for 2 hrs. The resultant products were separated on a 12% SDS PAGE gel with the undigested egg coat extracts loaded in adjacent lanes as controls. The bands that showed changed migration patterns after the PNGase F treatment are denoted by arrows. Click here for file

Additional file 6 **Figure S5.** Alternative spliced variants identified by thesequencing of the partial cDNAs obtained from the RT-PCR amplification of *B. belcheri* ovary transcripts, which suggests the presence of alternative splicing of BbZP transcripts, potentially contributing to the size heterogeneity observed in the PAGE gels in Figure 4a. It is noteworthy that the current study is not exhaustive because transcript variants of BbZP2, 3 and 5 had not been explicity searched for and not all of the spliced forms of BbZP1 and BbZP4 have been identified. Click here for file

Additional file 7 **Figure S6.** The amino acid alignment of the 4 Zona pellucida domain containing proteins from the non-jawed vertebrate, the sea lamprey (*P. marinus*). Click here for file

Additional file 8 **Figure S7.** The genomic organizations of the ZP protein genes in *B belcheri* and *B floridae* are highly similar. The BbZP genes are shown in blue squares, whereas the BfZP genes are in green. The trancriptional direction of each gene is indicated by arrows. The scaffold numbers where the gene(s) are localized are shown to the right of each synteny. Click here for file

Additional file 9 **Figure S8.** The raw mass-spectrometry data files of slices 1, 2 and 3 of the fertilized eggs. Click here for file
