# Supplementary material for: Proteomic characterization and evolutionary analyses of zona pellucida domain-containing proteins in the egg coat of the cephalochordate, Branchiostoma belcheri
Source: BMC Evol Biol. 2012 Dec 8;12:239. doi: 10.1186/1471-2148-12-239 (PMC3543715; doi:10.1186/1471-2148-12-239)
Supplement: Additional file 2 — Figure S1. The predicted amino acid sequences and the alignments of the five B. belcheri ZP proteins. The gray scale indicates the various numbers (3 to 5) of identical residues in the 5 aligned sequences. The cysteine residues that define the zona pellucida domains are boxed. Except for BbZP4, the other BbZP proteins contained 10 positionally conserved cysteine residues, suggesting a type II ZP domain. Regions with a double underline indicate domains of LDLa; those with the single underlines indicate vWFA domains, and the bold lines indicate the ZP modules. [file 1471-2148-12-239-S2.doc]

**Additional Fig. S1**

The predicted amino acid sequences and the alignments of the five *B. belcheri* ZP proteins. The gray scale indicates the various numbers (3 to 5) of identical residues in the 5 aligned sequences. The cysteine residues that define the zona pellucida domains are boxed. Except for BbZP4, the other BbZP proteins contained 10 positionally conserved cysteine residues, suggesting a type II ZP domain. Regions with a double underline indicate domains of LDLa; those with the single underlines indicate vWFA domains, and the bold lines indicate the ZP modules.

.

**BbZP1**

**BbZP2**

**BbZP3**

**BbZP4**

**BbZP5**

**BbZP1**

**BbZP2**

**BbZP3**

**BbZP4**

**BbZP5**

**BbZP1**

**BbZP2**

**BbZP3**

**BbZP4**

**BbZP5**

**BbZP1**

**BbZP2**

**BbZP3**

**BbZP4**

**BbZP5**

**BbZP1**

**BbZP2**

**BbZP3**

**BbZP4**

**BbZP5**

**BbZP1**

**BbZP2**

**BbZP3**

**BbZP4**

**BbZP5**

**BbZP1**

**BbZP2**

**BbZP3**

**BbZP4**

**BbZP5**

**BbZP1**

**BbZP2**

**BbZP3**

**BbZP4**

**BbZP5**

**BbZP1**

**BbZP2**

**BbZP3**

**BbZP4**

**BbZP5**

**BbZP1**

**BbZP2**

**BbZP3**

**BbZP4**

**BbZP5**

MASIPSLLVLVSTFVIPLAEGSHFRGGTISWLPLDAPSETSAVFRFNLGWKKMSATGTGCDTAALQ 66

MA---APVCRAGMLLKSVILTLFFK--------DLVLCATETVTI--------------------- 34

MA---A--LRGLLLLFTASLVLGYS--------QDVPNDIPAYAS--------------------- 32

MAGTVWAIRATCVLLLIVGVAIGYP--------SRGSKARNYIDEDKLREMLTRLVKARDVDRLLH 58

M--IP---VDKGWFCRSLSLLLFVF--------LANVASEDGLPHE-------------------- 38

GQTLVTTNEDPWECVSGCASSGTVFLANQDYYCTEFNVGQNWAKAFNSFAHDFRSTEDTYVVGYES 132

----------------------------------GVEKEGRQ--CVNGRCHSDGGDKEPAQ---SS 61

----------------------------------SDDDSGYLGALVDLLNNGLAGALEPSKRGTDE 64

AETAAHLNERRDEASGEGSGEPIVVEFPGEVKEGSGEGSGAFPGLVKGFFGTEGSGEGSGFPGNVK 124

----------------------------------QDNAESPRRIRASMFFQPAGEETNETREETDS 70

CCWLQIQNQQTGVFHN-------------------PLISLRTTVDLGFRSDNGKGNHAPATSMQTT 179

EG--LLKLLRDTAVH---------------------------SRWMGKRARFGEGRR--------- 89

DGGTDEDGGQEEGGT---------------------------DEDGGEEEGSDEGPKE------VP 97

EGSGDEGSGETGGIEGFPGDVRGYGFPSYGLGGGGYYGLDSLVESSGEGSGFPGGVKEGGSEEGSG 190

RDRLMETAAARGRAN--------------------------------ERKVLG------------- 91

IRVTNGCLAPDYPAGKLAVADIDGDTVKCRFAQGATECGEACDQFPGLVLNEDCTYSYNGPASITI 245

----YWKNVVCNDREVVPSLWVCDGTADCMNSEDESGCLGYCGDYGYPVPGCECDVGFCMNDRPSP 151

APTEKAPTFDCDANQTVSFNWRCDGEEDCANGKDEEDCAGFCGDNGYPSAECACDK--CNSARNSP 161

EDQEDEPVQQCPDGDFISASWRCDGTTDCNDGSDEQGCEELCSGLGYPG-DRDCRCASCKADRPSQ 256

---VFAREYRCGDGETIPQLWICDGTEDCRNGNDEENCADRCGXLGYPXLGCECDIGYCAAQRPSP 154

SSGTSLFFVVAVMVEDFPVQTIFRHNQEVPTTQALSSVPLQFLIEVKNDPGVLCGSEPVLTGATPA 311

VCACGFRDCLFPLPRSCRRPTFLELPAFHPSAIGLKQFQ-------EPECQVEMDLIFLLDGSASV 210

VCACPFADCESDLPESCDGAETFDEPLFGLSALGSKHPYGGR---VAAPCYETIQLFFVADGSASV 224

NCACPTSQCEDVLPDKCDQLTILATPPFDPTNAALKHHYPGTGTGRVTGGAMRADMVFITDGSASI 321

LCRCQPGQCRAQIPRKCDEAPIFPAPPFNPFLLASRHRTS------VAECSTPVDLAFVMDGSASV 214

QDTCLPIPDSVEYTMTVEAQPVAPATLAEINVLGPKGMTKSALTVTGDISSTTITWTPAPGQRGPH 377

G------------HLNFEKEKKFCRQLVSDFDIGPNKTRVATIQYSIEQQ--------------DE 250

G------------AMNFEQVKKFMSDAVDSFVIGPGHTTAAVIQYAYDYR--------------HE 264

G------------TFNFEEIKKFMREMVEGLTVSPSSFRVGAMQFAYENR--------------EE 361

G------------PLEFEKSKKFVRDVVDGFEVGPTQTRVGVVQFAWMVQ--------------AE 254

IVCFYAEDSNAVQSDRTCVSLMVGGSITPPAVDAPTLIPTPGTAPTDYPCDTTLRFSATFDQLVLP 443

FRF-DLPDVHTLRNALGQIVYMDG----------------PGTETGKAIMYMASRFPQR------- 292

IYLGQYQDSVSLKNAIACIQYLDG----------------GGTQTAAAINAMVNAAIQVP------ 308

FGLEDHHDNAGVDAAICAIPYMDG----------------PGTYTGEAILFAKDFMFAPIR----- 406

FHLGDYLDGTDLRNAIARIRYMDG----------------PGTEIGKALVFTKRRLFSELYGARPE 304

PTSDTFIIFYDSTNAEFYKHNTKIPDGQPAPAQSNTYVFDVPANTFQPLGTYTIAMQTGALEGVTG 509

-EGAKKIAIVITDGKNNPESRVSVRMAADYAREDGITLYAVGVGT---------EVDIRELTDLAG 348

-TTGKRMGMVITDGRSQ-AGRTTVLDASNYARDNGFTMYAVGVG----------NADSAEMLQIAG 362

-PEIRHIGIVITDGKTS-LGAMDVGTASHSAQQAGIVMYAIGIGLMH------DATYNAQLQAIAG 464

TQGVPRVIILITDGRSSPESQISVWTAAQELHSAGVVVYAVGVGT---------AVDEAELETAAS 361

CGAGAGVQSDAYETAG-------------------------------------------------- 525

DPTRVYNVEDFQSLS--------------------------------------------------- 363

DSSRVMHVQDHSQLV--------------------------------------------------- 377

PSGKVFHVGDFSQLSGIISALLADIFALPPPIIAPAPAPAPYPVPAPAPYPVPAPAPYPVPAPAPY 530

DSSKVYHVRDFDSLM--------------------------------------------------- 376

-----------------------GTWSFTCKFYIPAPVTTAPPAPPPATTAATPGATVRVVGTPAP 568

----------------------------AFAEQGPLQTELCLSEPITPTDNQTSGPSGFPGISRSG 401

----------------------------FETLRPQIQGDFCPDY---------------------- 393

PVPAPYPVPAPAPYPVPAPAPYPVPAPAPYPVPAPAPYPVPAPAPYPVPAPAPAPAPYPVPAPAPY 596

----------------------------MLSLQDSIQQSVCLGG---------------------- 392

Additional Figure S1 continued

**BbZP1**

**BbZP2**

**BbZP3**

**BbZP4**

**BbZP5**

**BbZP1**

**BbZP2**

**BbZP3**

**BbZP4**

**BbZP5**

**BbZP1**

**BbZP2**

**BbZP3**

**BbZP4**

**BbZP5**

**BbZP1**

**BbZP2**

**BbZP3**

**BbZP4**

**BbZP5**

**BbZP1**

**BbZP2**

**BbZP3**

**BbZP4**

**BbZP5**

**BbZP1**

**BbZP2**

**BbZP3**

**BbZP4**

**BbZP5**

GALPTMPVTAIVVTCSPTSITVTIPLSEITGVNAADIRYRSAPCLPVVSGDSVSITTGFQECGTTM 634

PISSEDLRTYAIISCSSFSMQVDFPRMYFPQVYAPALHLADPSCTARGNSTHVSILAPLVGCGTTS 467

--------TNWTISCNGDSMTVAILRSRMPSLSATDLHLTDPSCGATDNGTHLVLTSPLTGCGTSS 399

PVPAPAPAPSVGVSCSDSTLEIDLAASSFPTVRTSNLRLVDPACRATSNGTHMIMKSPLNACGTTS 662

--------ANVTVTCTSYHMQVDLSRAQLPNLEARRMRVIDPSCRATSNATHVTLWTYLDSCGTSR 450

TTQGDELVYENEIHT-------EFTSTAIRGNSVDGKVECAYDSNTVVTGK--QFSALMGSVFGRR 691

SMTADFILYRNKVIENGDMLPGASTSPIIRACGFELDFTCQMPRHKSVLTG--YNPVLQPDRFYEQ 531

SEQPNNIVYRNRVTQ--DWKRRAHSEPIIRDCGFILDFSCELPRHKTVVAD--YNPIVQPDRYSER 513

YETPDFIVYDNVVTD----ESLARAGEIIRDCGFEMRIQCQIPRRLTVSGD--FDPVQVPEMYSAV 722

TETEDAIVYSNTVVEDLDLVPAPTGGSPAPQCGLQVRFTCRMARTGTVATTGAFNAITEPERFYST 516

SSGQFEFSFDFYTDNTFSTAFTSYPVSYHPNQEMFIGVHLVSTNTDLVLFADNCKATPG-VEWDSS 756

GTGELQAVLRFCRDASCLSYNTDSPLVVKIGGDVIVEIELLTSDPDLSIMVEDCITTDTPTSTGGG 597

GNGHIHAILRFCREPGCPAYITEYPVMARVCEDIYVEIQLITADPDLSMLTERCVARDTPTVATGT 579

GTGDINVIMHFCVDSVCSQYKP-YPVTYRVCEQVYVEVQLLASDPDLSILVLTGEATTS-MARTGG 786

GHGNFHLTMTFYTDG-SYQVPAREPLSVRVCDMVYVQIQLYSADPDLTVFALDCWATPTAEDTAGP 581

PSYTIRQNGCNVDPLLTWYEPADAARAAREENFGIVVFRFADYST--LYIHCDVVVCAAADTSSYC 820

YQYQITQEGCGVDQTYHQLAAPRHSVDR----FKFQAFNFVSDVT-RVYLTCGVLVCRASQPGNRC 658

TVYELVRDGCPVDPTYHELAAPNHATDR----FKFEAFKFVTDFP-KVYLTCDLLVCKASDPANRC 640

PLYDIINNGCGVDPTYVAYPAPNHAVIR----FGFQAFKFATDYP-KVYLHADVLICKSSAIGNRC 847

LLYDIXKDGCXVDPTFQAGTSGIPSAQR----FGFQAFKFAGASSGSVVLRCQVLVCVASDKDSRC 643

ATAPASCGT-----GRKRRDADVVDKLHRFEVTSGPVKIIRDDGGHNNNRQAHGDWKEAFSAVVSP 881

AHG---CVQHWV—-GKR--SVVNLDDIMIHVTSGPLVLLLPTTPRP-------------------- 697

AQG---CQRMVIPIGKRKRSADDRMELRRTVVSGPLVMEIDGDRKYMDTI---------------- 687

SQG---CGTA----GRRRRDTSVTPGVALYRVTAPAPMILFDDVDETLVPQ--------------- 891

AQG---CGHVS---RREARDDVISXQGXSLVXGPIVLTKDD------------------------- 678

PVLGVLMVCLGVIFLSAAVSCYYLRRYQQLYTTVFETDHLKPSILS 927

----------------------------------------------

----------------------------------------------

----------------------------------------------

----------------------------------------------
