# Supplementary material for: Proteomic characterization and evolutionary analyses of zona pellucida domain-containing proteins in the egg coat of the cephalochordate, Branchiostoma belcheri
Source: BMC Evol Biol. 2012 Dec 8;12:239. doi: 10.1186/1471-2148-12-239 (PMC3543715; doi:10.1186/1471-2148-12-239)
Supplement: Additional file 3 — Figure S2. The amino acid sequence alignment of the von Willebrand factor type A (vWFA) domains from the BbZPs (BbZP2, 3 4, and 5) and two ZP proteins of Ciona Intestinalis (XP_002120027.1 and XP_002127158.1). [file 1471-2148-12-239-S3.doc]

**Additional Fig. S2**

The amino acid sequence alignment of the von Willebrand factor type A (vWFA) domains from the BbZPs (BbZP2, 3 4, and 5) and two ZP proteins of *Ciona Intestinalis* (XP_002120027.1 and XP_002127158.1).

**BbZP2**

**BbZP3**

**BbZP4**

**BbZP5**

**XP_002120027.1**

**XP_002127158.1**

**BbZP2**

**BbZP3**

**BbZP4**

**BbZP5**

**XP_002120027.1**

**XP_002127158.1**

**BbZP2**

**BbZP3**

**BbZP4**

**BbZP5**

**XP_002120027.1**

**XP_002127158.1**

EMDLIFLLDGSASVGHLNFEKEKKFCRQLVSDFDIGPNKTRVATIQYSIEQQDEFRF-DLPDVHT

TIQLFFVADGSASVGAMNFEQVKKFMSDAVDSFVIGPGHTTAAVIQYAYDYRHEIYLGQYQDSVS

RADMVFITDGSASIGTFNFEEIKKFMREMVEGLTVSPSSFRVGAMQFAYENREEFGLEDHHDNAG

PVDLAFVMDGSASVGPLEFEKSKKFVRDVVDGFEVGPTQTRVGVVQFAWMVQAEFHLGDYLDGTD

SVAARFGECG-IQVEQRE--NSYMFKQQVVWN----MISTSVERPTVLVDITCNYTRDYAVLAGP

SILPPFSNCG-TQVIQRGPGDDYTFSNTVVWNREVNNTQNIIDREMILLDFKCIYEDTYTVSGPS

LRNALGQIVYMDGPGTETGKAIMYMASRFPQR--------EGAKKIAIVITDGKNNPESRVSVRM

LKNAIACIQYLDGGGTQTAAAINAMVNAAIQVP-------TTGKRMGMVITDGRSQ-AGRTTVLD

VDAAICAIPYMDGPGTYTGEAILFAKDFMFAPIR------PEIRHIGIVITDGKTS-LGAMDVGT

LRNAIARIRYMDGPGTEIGKALVFTKRRLFSELYGARPETQGVPRVIILITDGRSSPESQISVWT

IIPTVNKVDFQTSYGVFTVQMELYRNQ-------------------------------------Q

LLPTLNVLKFQTGQGRFEVRMGLFDDN-------------------------------------S

AADYAREDGITLYAVGVGT---EVDIRELTDLAGDPTRVYNVEDFQSLS

ASNYARDNGFTMYAVGVG----NADSAEMLQIAGDSSRVMHVQDHSQLV

ASHSAQQAGIVMYAIGIGLMHDATYNAQLQAIAGPSGKVFHVGDFSQLS

AAQELHSAGVVVYAVGVGT---AVDEAELETAASDSSKVYHVRDFDSLM

FSEDAKFGRQLMIPIEQEV----CVEQSLINVMPDHLVLSLLRCWASTV

YSGSHEYTQQPTISIGTYV----YVQVELAHVSDPHLVVTMERCFASQS
