# Supplementary figures and images for: Proteomic characterization and evolutionary analyses of zona pellucida domain-containing proteins in the egg coat of the cephalochordate, Branchiostoma belcheri
Source: BMC Evol Biol. 2012 Dec 8;12:239. doi: 10.1186/1471-2148-12-239 (PMC3543715; doi:10.1186/1471-2148-12-239)

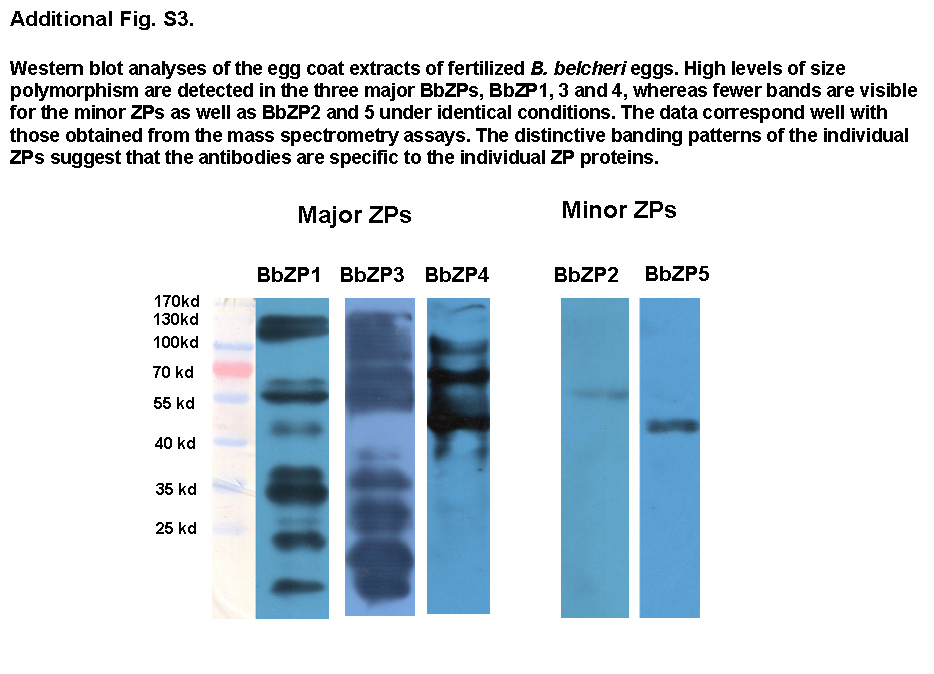

Supplement: Additional file 4 — Figure S3. Western blot analysisof the egg coat extracts of fertilized B. belcheri eggs. High levels of size polymorphism are detected in the three major BbZPs, BbZP1, 3 and 4, whereas fewer bands are visible for the minor ZPs as well as BbZP2 and 5 under identical conditions. The data correspond well with those obtained from the mass spectrometry assays. The distinctive banding patterns of the individual ZPs sggests that the antibodies are specific to the individual ZP proteins. [file 1471-2148-12-239-S4.jpeg]

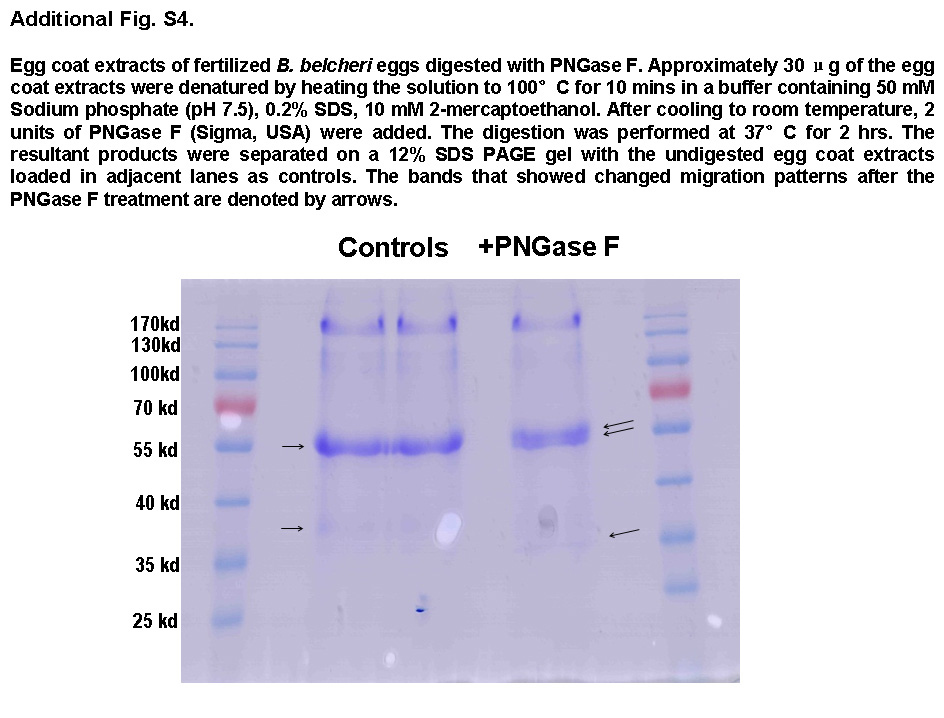

Supplement: Additional file 5 — Figure S4. Eggcoat extracts of fertilized B. belcheri eggs digested with PNGase F. Approximately 30 μ g of the egg coat extracts were denatured by heating the solution to 100°C for 10 minutes in a buffer containing 50 mM Sodium phosphate (pH 7.5), 0.2% SDS , 10 mM 2.mercaptoethanol. After cooling the room temperature, 2 units of PNGase F (Sigma USA) were added. The digestion was performed at 37°C for 2 hrs. The resultant products were separated on a 12% SDS PAGE gel with the undigested egg coat extracts loaded in adjacent lanes as controls. The bands that showed changed migration patterns after the PNGase F treatment are denoted by arrows. [file 1471-2148-12-239-S5.jpeg]

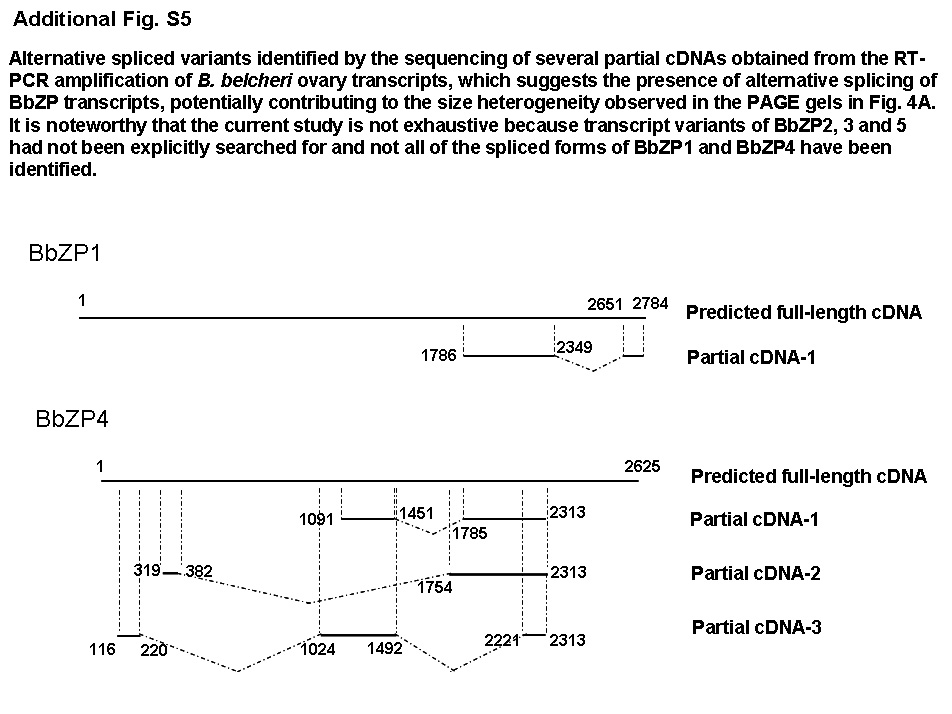

Supplement: Additional file 6 — Figure S5. Alternative spliced variants identified by thesequencing of the partial cDNAs obtained from the RT-PCR amplification of B. belcheri ovary transcripts, which suggests the presence of alternative splicing of BbZP transcripts, potentially contributing to the size heterogeneity observed in the PAGE gels in Figure 4a. It is noteworthy that the current study is not exhaustive because transcript variants of BbZP2, 3 and 5 had not been explicity searched for and not all of the spliced forms of BbZP1 and BbZP4 have been identified. [file 1471-2148-12-239-S6.jpeg]

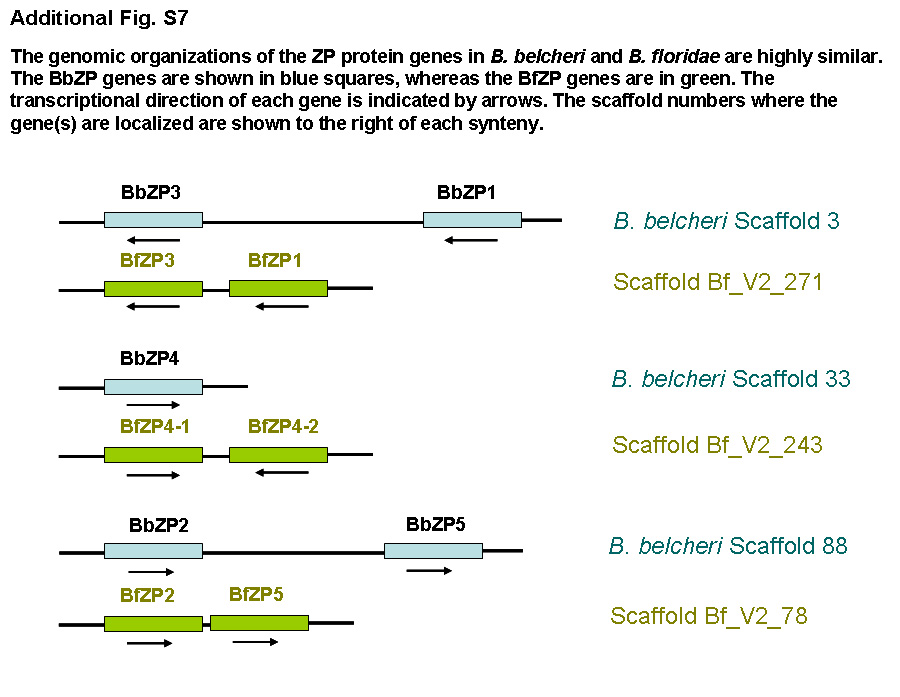

Supplement: Additional file 8 — Figure S7. The genomic organizations of the ZP protein genes in B belcheri and B floridae are highly similar. The BbZP genes are shown in blue squares, whereas the BfZP genes are in green. The trancriptional direction of each gene is indicated by arrows. The scaffold numbers where the gene(s) are localized are shown to the right of each synteny. [file 1471-2148-12-239-S8.jpeg]
