# Supplementary material for: Proteomic characterization and evolutionary analyses of zona pellucida domain-containing proteins in the egg coat of the cephalochordate, Branchiostoma belcheri
Source: BMC Evol Biol. 2012 Dec 8;12:239. doi: 10.1186/1471-2148-12-239 (PMC3543715; doi:10.1186/1471-2148-12-239)
Supplement: Additional file 7 — Figure S6. The amino acid alignment of the 4 Zona pellucida domain containing proteins from the non-jawed vertebrate, the sea lamprey (P. marinus). [file 1471-2148-12-239-S7.doc]

Additional Fig. S6

The amino acid alignment of the 4 Zona pellucida domain containing proteins from the non-jawed vertebrate, the sea lamprey (*P. marinus*).

**Lamprey-zp4-1**

**Lamprey-zp4-2**

**Lamprey-zp2**

**Lamprey-zpAX**

**Lamprey-zp4-1**

**Lamprey-zp4-2**

**Lamprey-zp2**

**Lamprey-zpAX**

**Lamprey-zp4-1**

**Lamprey-zp4-2**

**Lamprey-zp2**

**Lamprey-zpAX**

**Lamprey-zp4-1**

**Lamprey-zp4-2**

**Lamprey-zp2**

**Lamprey-zpAX**

**Lamprey-zp4-1**

**Lamprey-zp4-2**

**Lamprey-zp2**

**Lamprey-zpAX**

**Lamprey-zp4-1**

**Lamprey-zp4-2**

**Lamprey-zp2**

**Lamprey-zpAX**

**Lamprey-zp4-1**

**Lamprey-zp4-2**

**Lamprey-zp2**

**Lamprey-zpAX**

**Lamprey-zp4-1**

**Lamprey-zp4-2**

**Lamprey-zp2**

**Lamprey-zpAX**

**Lamprey-zp4-1**

**Lamprey-zp4-2**

**Lamprey-zp2**

**Lamprey-zpAX**

-----VDASESSACASTLPNVR---------CGAAAARSDRDACSRRGCCYDP----IRQVCL 45

---------------------------------------------------------------

FLKVTVSRILPPFSASTLHDKRPMSWYLDIDNGTAQWSVPVAYASQAGYVFDSQELTLTVTAL 63

---------------------------------------------------------LSVSCQ 6

YGEAAIYIHIGHIYMASITVINRVN-------------------------------------- 70

---------------------------------------------------------------

YNASGVFYSQRNVHCGSVKLIYQVAPPPPPLRVARAIATCPGYLQCNDTSLVVTVPPLPGPLD 126

FPTTDVVACFPNGTVAVAAPKGHTKP------------------------------------- 32

------------------FSSTIAFSCLLW-LVLSMP-------------------------- 88

---------------------------------------------------------------

AVKVGTTPVYPGPSSVPGVEVRLENSGGLWGFVLSLRRTSPLVGVEPMVLWKPWNTQVRFGGD 189

-------------------------EVDLWRLALRER-------------------------- 44

--------------------------------------------------HFVSCTCTMCTLY 101

---------------------------------------------------------------

GRGELAVIYKKNCEASVVSVVAECSTDGQMTFEVYASSTRPPLELATVHVRDPSCLPVVVTAE 252

-----------------------------------------------------SCGPVYVNTT 54

FAVYHFSVNSCGVTTRWNEVELVYGALVTAEHELCIDGDVAITKSSTFKLLLECRLLRTDDRP 164

--------------------------------------------------------------L 1

KAVFVVPLDACGTTQQMVDGKLVYENEAVSLMRDSIH--VIISRSSEYRMKITCEFS-GDDLL 312

KAYFVFTVSSCGTSRTIEGAFLVYENEISLHIQWFALNVSKFCTVPLQRLKVICRYR-INDTV 116

FLGVQVNTYPPPLPATAIGTLFIELQVATDGDYLDYYS--QFPVVKFLRDPIFLEVRLLDHPD 225

QLGVTVATLPPPRPVTGAGPLYLELRIARDGRYLDYYS--QFPVVKFLRDPIFLEVRLLDHPD 62

MLGVTVPTLSPPSPANGTGPLVIELTMFPDVDYVAPYVAADYPLVRFLREPLFVQVQLLAHPD 375

VISSVVQARRGPTADEQLVPVPIVMKISRDADYTSYYLDEEYPVVSYLQEPLYFEVRLLGLED 179

PSLVLVLQDCWATPTPDPLNS-VQWRVLDD--------HCPFTGDNFPTVLHPMTEFPDVPLAT 280

PNLVLVLQDCWATPTPDPLNS-VQWRVLDN--------HCPFSGDDYQTVLHPMSEFPSVLLSS 117

PVLELRLADCWATSQPDP-NSLPQWDLLVS--------GCPFSGDNYLSTIHTVSP-ASVPLHL 430

QSIEMFLENCWATNLPTA-DSQPRWDIIVNRSTHGPATSCENANDNYLTVFHPVPHNSSVPFPQ 242

HRKRLEVKTFTFAGPQGNVFPNGQLYFHCRAYVCQG---DDRSCVPT-CHLSRLKS-KS---- 334

HYKRFEVKTFVFTGPQDAMVPYGKLYFHCSAYVCKGG--DESSCVPG-CSSKFKRAR------ 171

RFKRFEVKTFVFADPAPLGALRDTLYFHCSATVCDSNSADLPQCGPSSCSPSTRRARRDIDQA 492

NVKRFEVKTFVFEMDIGNDFQG-QLFFHCSVVVCDAVNFDD-VCQMD-CIPNRRRVGKS---- 298

---------------LFKTTAREPRS-------------------------------------- 345

---------------VTQNPTPSTST-------------------------------------- 182

VENGMTHVVSLAGPIVFTTDASKSRPSVSAATPTGGVWVPGLLAAACSVLAVSIAVALVAKLLC 556

---------------TLVLDL-K-RS-------------------------------------- 307
