# Supplementary material for: Proteomic characterization and evolutionary analyses of zona pellucida domain-containing proteins in the egg coat of the cephalochordate, Branchiostoma belcheri
Source: BMC Evol Biol. 2012 Dec 8;12:239. doi: 10.1186/1471-2148-12-239 (PMC3543715; doi:10.1186/1471-2148-12-239)
Supplement: Supplementary file 5 [file 1471-2148-12-239-S1.doc]

Other non-ZP proteins identified from *B. belcheri* fertilized egg coats

Protein ID	Highest spectra counts identified	Description
093340_PRF0	87	multiple EGF-like domains protein 6-like
256590_PRF0	56	vitellogenin
278170_PRF0	37	vitellogenin
256510_PRF0	20	zonadhesin-like
256600_PRF0	18	vitellogenin
131700_PFF0	18	melanotransferrin-like
093130_PFF0	12	matrilin-2
092060_PFF0	10	similar to apolipoprotein B
